# Healthcare worker perceptions about infection prevention and control processes and practices in Latin America

**DOI:** 10.1017/ash.2023.327

**Published:** 2023-09-29

**Authors:** Valeria Fabre, Pilar Beccar-Varela, Carolyn Herzig, Guadalupe Reyes-Morales, Clare Rock, Rodolfo Quiros

## Abstract

**Background:** The burden of hospital-associated infections (HAIs) and antimicrobial resistance (AMR) in Latin America is high. Improving engagement by healthcare workers (HCWs) in infection prevention and control (IPC) may lead to better patient outcomes; however, little is known about HCW perceptions of IPC in the region. We sought to understand HCW perceptions of IPC processes and practices. **Methods:** During August–September 2022, HCWs from 30 hospitals with IPC programs in 4 Latin American countries (Panama, Guatemala, Ecuador, and Argentina) were invited to participate in an electronic, voluntary, anonymous survey about their perceptions of IPC at their hospitals. Physicians, nurses, and environmental care (EVC) personnel were prioritized for recruitment. All respondents were asked 18 questions; IPC team members were asked 5 additional questions about specific activities implemented by IPC programs, how data are used, and how IPC could be improved. Answers with 5-point Likert scale responses were categorized into 2 groups (eg, strongly agree or agree vs neutral, disagree, or strongly disagree) for analysis. **Results:** Of 1,252 HCWs who completed the survey, 181 (14%) were IPC team members, 1,095 (87%) had direct patient contact, and 1,156 (92%) worked >20 hours per week. Figure 1 shows participant characteristics. Most participants (56%) rated their IPC program as very good, 38% rated it as good, and 6% rated it as bad. Physicians were less likely to give a favorable rating. Compliance with prevention bundles and hand hygiene (HH) by colleagues was rated as poor by 28% and 22% of HCWs, respectively; however, only 11% and 5% indicated that their own compliance was poor, respectively. Also, 25% of participants reported not receiving or only occasionally receiving HH compliance data. Similarly, 41% of participants reported not receiving HAI data on a regular basis, and 19% of IPC nurses reported not receiving data despite being responsible for conducting surveillance. Furthermore, 41% of respondents indicated not receiving or only occasionally receiving IPC training or education relevant to their role. When asked about the safety climate, 16% of participants reported not feeling appreciated. In addition, 22% of IPC nurses and 37% of individuals in the “other” category (eg, health technicians and therapists) were more likely to report this. When IPC team members were asked how frequently specific activities were conducted (Fig. 2), several opportunities for improvement were identified, including improving HCW access to HH data and development of strategic plans. **Conclusions:** Improving HCW access to training on IPC and to data on HAI burden and compliance with HH and prevention bundles should be emphasized in Latin American hospitals.

**Disclosures:** None

